# Supplementary material for: Smooth Interpolating Curves with Local Control and Monotone Alternating Curvature
Source: Comput Graph Forum. 2022 Oct 6;41(5):25–38. doi: 10.1111/cgf.14600 (PMC9827861; doi:10.1111/cgf.14600)
Supplement: Supplementary file 1 — Supplement Material [file CGF-41-25-s001.zip › Local-Smooth-Interpolating-MonoCurvature/extern/clothoids/submodules/quarticRootsFlocke/docs/api-cpp/program_listing_file_PolynomialRoots-2-Cubic.cc.html]

Program Listing for File PolynomialRoots-2-Cubic.cc — Quartic Roots v1.0.0

### Navigation

- index
- toc
- Quartic Roots »
- Program Listing for File PolynomialRoots-2-Cubic.cc

# Program Listing for File PolynomialRoots-2-Cubic.cc¶

↰ Return to documentation for file (`PolynomialRoots-2-Cubic.cc`)

```
/*--------------------------------------------------------------------------*\
 |                                                                          |
 |  Copyright (C) 2017                                                      |
 |                                                                          |
 |         , __                 , __                                        |
 |        /|/  \               /|/  \                                       |
 |         | __/ _   ,_         | __/ _   ,_                                |
 |         |   \|/  /  |  |   | |   \|/  /  |  |   |                        |
 |         |(__/|__/   |_/ \_/|/|(__/|__/   |_/ \_/|/                       |
 |                           /|                   /|                        |
 |                           \|                   \|                        |
 |                                                                          |
 |      Enrico Bertolazzi                                                   |
 |      Dipartimento di Ingegneria Industriale                              |
 |      Universita` degli Studi di Trento                                   |
 |      email: enrico.bertolazzi@unitn.it                                   |
 |                                                                          |
\*--------------------------------------------------------------------------*/

#ifdef __GNUC__
#pragma GCC diagnostic ignored "-Wunused-function"
#endif
#ifdef __clang__
#pragma clang diagnostic ignored "-Wglobal-constructors"
#pragma clang diagnostic ignored "-Wvla-extension"
#pragma clang diagnostic ignored "-Wvla"
#pragma clang diagnostic ignored "-Wunused-function"
#pragma clang diagnostic ignored "-Wdocumentation-unknown-command"
#endif

#include "PolynomialRoots.hh"

#include <cmath>
#include <iostream>
#include <algorithm>
#include <limits>

#ifndef DOXYGEN_SHOULD_SKIP_THIS

#define MAX_ITER_SAFETY 50

namespace PolynomialRoots {

  using std::abs;
  static valueType const machepsi = std::numeric_limits<valueType>::epsilon();
  static valueType const third    = 1./3.;
  static valueType const one27th  = 1./27.;
  static valueType const two27th  = 2./27.;

  indexType
  Cubic::getRealRoots( valueType r[] ) const {
    indexType nr = 0;
    if ( cplx ) {
      if ( nrts > 2 ) r[nr++] = r2;
    } else {
      if ( nrts > 0 ) r[nr++] = r0;
      if ( nrts > 1 ) r[nr++] = r1;
      if ( nrts > 2 ) r[nr++] = r2;
    }
    return nr;
  }

  indexType
  Cubic::getPositiveRoots( valueType r[] ) const {
    indexType nr = 0;
    if ( cplx ) {
      if ( nrts > 2 && r2 > 0  ) r[nr++] = r2;
    } else {
      if ( nrts > 0 && r0 > 0 ) r[nr++] = r0;
      if ( nrts > 1 && r1 > 0 ) r[nr++] = r1;
      if ( nrts > 2 && r2 > 0 ) r[nr++] = r2;
    }
    return nr;
  }

  indexType
  Cubic::getNegativeRoots( valueType r[] ) const {
    indexType nr = 0;
    if ( cplx ) {
      if ( nrts > 2 && r2 < 0  ) r[nr++] = r2;
    } else {
      if ( nrts > 0 && r0 < 0 ) r[nr++] = r0;
      if ( nrts > 1 && r1 < 0 ) r[nr++] = r1;
      if ( nrts > 2 && r2 < 0 ) r[nr++] = r2;
    }
    return nr;
  }

  indexType
  Cubic::getRootsInRange( valueType a, valueType b, valueType r[] ) const {
    indexType nr = 0;
    if ( cplx ) {
      if ( nrts > 2 && r2 >= a && r2 <= b ) r[nr++] = r2;
    } else {
      if ( nrts > 0 && r0 >= a && r0 <= b ) r[nr++] = r0;
      if ( nrts > 1 && r1 >= a && r1 <= b ) r[nr++] = r1;
      if ( nrts > 2 && r2 >= a && r2 <= b ) r[nr++] = r2;
    }
    return nr;
  }

  indexType
  Cubic::getRootsInOpenRange( valueType a, valueType b, valueType r[] ) const {
    indexType nr = 0;
    if ( cplx ) {
      if ( nrts > 2 && r2 > a && r2 < b ) r[nr++] = r2;
    } else {
      if ( nrts > 0 && r0 > a && r0 < b ) r[nr++] = r0;
      if ( nrts > 1 && r1 > a && r1 < b ) r[nr++] = r1;
      if ( nrts > 2 && r2 > a && r2 < b ) r[nr++] = r2;
    }
    return nr;
  }

  void
  Cubic::eval( valueType x, valueType & p, valueType & dp ) const {
    valueType const & A = ABCD[0];
    valueType const & B = ABCD[1];
    valueType const & C = ABCD[2];
    valueType const & D = ABCD[3];
    if ( std::abs(x) > 1 ) {
      valueType x2 = x*x;
      valueType x3 = x2*x;
      p  = (((D/x+C)/x+B)/x+A)*x3;
      dp = ((C/x+2*B)/x+3*A)*x2;
    } else {
      p  = ((A*x+B)*x+C)*x+D;
      dp = (3*A*x+2*B)*x+C;
    }
  }

  // - - - - - - - - - - - - - - - - - - - - - - - - - - - - - - - - - - - - - -
  static
  inline
  valueType
  guess1( valueType const a[3] ) {
    valueType const p =  1.09574;
    valueType const q = -3.239E-1;
    valueType const r = -3.239E-1;
    valueType const s =  9.57439E-2;
    return p+q*a[1]+r*a[2]+s*a[1]*a[2];
  }

  static
  inline
  valueType
  guess2( valueType const a[3] ) {
    valueType const p = -1.09574;
    valueType const q =  3.239E-1;
    valueType const r = -3.239E-1;
    valueType const s =  9.57439E-2;
    return p+q*a[1]+r*a[2]+s*a[1]*a[2];
  }

  static
  inline
  valueType
  guess3( valueType const a[3] ) {
    valueType const p =  1.14413;
    valueType const q = -2.75509E-1;
    valueType const r = -4.45578E-1;
    valueType const s = -2.59342E-2;
    valueType t = a[2]/3;
    if ( a[0] < t*(2*t*t-1) ) return  p+q*a[0]+r*a[2]+s*a[0]*a[2];
    else                      return -p+q*a[0]+r*a[2]-s*a[0]*a[2];
  }

  static
  inline
  valueType
  guess4( valueType const a[3] ) {
    valueType const q = -7.71845E-1;
    valueType const s = -2.28155E-1;
    if ( a[0] > 0 ) return (q+s*a[2])*a[0];
    else            return (q-s*a[2])*a[0];
  }

  static
  inline
  valueType
  guess5( valueType const a[3] ) {
    valueType p, q, r, s;
    valueType tmp = two27th-a[1]/3;
    if ( a[1] <= third ) {
      if ( a[0] < tmp ) {
        p =  8.78558E-1;
        q = -5.71888E-1;
        r = -7.11154E-1;
        s = -3.22313E-1;
      } else {
        p = -1.92823E-1;
        q = -5.66324E-1;
        r = +5.05734E-1;
        s = -2.64881E-1;
      }
    } else {
      if ( a[0] < tmp ) {
        p = 1.19748;
        q = -2.83772E-1;
        r = -8.37476E-1;
        s = -3.56228E-1;
      } else {
        p = -3.45219E-1;
        q = -4.01231E-1;
        r =  2.07216E-1;
        s = -4.45532E-3;
      }
    }
    return p+q*a[0]+r*a[1]+s*a[0]*a[1];
  }

  static
  inline
  valueType guess6( valueType const a[3] ) {
    valueType p, q, r, s;
    valueType tmp = a[1]/3-two27th;
    if ( a[1] <= third ) {
      if ( a[0] > tmp ) {
        p = -8.78558E-1;
        q = -5.71888E-1;
        r =  7.11154E-1;
        s = -3.22313E-1;
      } else {
        p =  1.92823E-1;
        q = -5.66324E-1;
        r = -5.05734E-1;
        s = -2.64881E-1;
      }
    } else {
      if ( a[0] > tmp ) {
        p = -1.19748;
        q = -2.83772E-1;
        r =  8.37476E-1;
        s = -3.56228E-1;
      } else {
        p =  3.45219E-1;
        q = -4.01231E-1;
        r = -2.07216E-1;
        s = -4.45532E-3;
      }
    }
    return p+q*a[0]+r*a[1]+s*a[0]*a[1];
  }

  /*
  ||   _   _               _              ____  _               _   _
  ||  | \ | | _____      _| |_ ___  _ __ | __ )(_)___  ___  ___| |_(_) ___  _ __
  ||  |  \| |/ _ \ \ /\ / / __/ _ \| '_ \|  _ \| / __|/ _ \/ __| __| |/ _ \| '_ \
  ||  | |\  |  __/\ V  V /| || (_) | | | | |_) | \__ \  __/ (__| |_| | (_) | | | |
  ||  |_| \_|\___| \_/\_/  \__\___/|_| |_|____/|_|___/\___|\___|\__|_|\___/|_| |_|
  */

  // x^3 + a * x^2 + b * x + c
  static
  indexType
  NewtonBisection(
    valueType   a,
    valueType   b,
    valueType   c,
    valueType & x
  ) {
    valueType p, dp;
    evalMonicCubic( x, a, b, c, p, dp );
    valueType t = p; // save p(x) for sign comparison
    x -= p/dp; // 1st improved root

    indexType iter       = 1;
    indexType oscillate  = 0;
    indexType nconverged = 0;
    bool      bisection  = false;
    bool      converged  = false;
    valueType s(0), u(0); // to mute warning
    while ( ! ( nconverged > 1 || bisection ) && iter < MAX_ITER_SAFETY ) {
      ++iter;
      evalMonicCubic( x, a, b, c, p, dp );
      if ( p*t < 0 ) { // does Newton start oscillating ?
        if ( p < 0 ) {
          ++oscillate; // increment oscillation counter
          s = x;       // save lower bisection bound
        } else {
          u = x; // save upper bisection bound
        }
        t = p; // save current p(x)
      }
      dp = p/dp; // Newton correction
      x -= dp;   // new Newton root
      bisection = oscillate > 2; // activate bisection
      converged = std::abs(dp) <= (1+std::abs(x)) * machepsi; // Newton convergence indicator
      if ( converged ) ++nconverged; else nconverged = 0;
    }
    if ( bisection ) {
      t = u - s; // initial bisection interval
      while ( std::abs(t) > (1+std::abs(x)) * machepsi && iter < MAX_ITER_SAFETY ) { // bisection iterates
        ++iter;
        p = evalMonicCubic( x, a, b, c );
        if ( p < 0 ) s = x;
        else         u = x; // keep bracket on root
        t = (u-s)/2; // new bisection interval
        x = s + t;   // new bisection root
      }
    }
    return iter;
  }

  /*\
   *  Calculate the zeros of the cubic a*z^3 + b*z^2 + c*z + d.
  \*/

  void
  Cubic::findRoots() {
    valueType const & A = ABCD[0];
    valueType const & B = ABCD[1];
    valueType const & C = ABCD[2];
    valueType const & D = ABCD[3];
    nrts = iter = 0;
    cplx = dblx = trpx = false;
    // special cases
    if ( isZero(A) ) {
      Quadratic qsolve( B, C, D );
      nrts = qsolve.numRoots();
      cplx = qsolve.complexRoots();
      dblx = qsolve.doubleRoot();
      r0   = qsolve.real_root0();
      r1   = qsolve.real_root1();
      return;
    }
    if ( isZero(D) ) {
      Quadratic qsolve( A, B, C );
      nrts = qsolve.numRoots()+1;
      cplx = qsolve.complexRoots();
      r0   = qsolve.real_root0();
      r1   = qsolve.real_root1();
      r2   = 0;
      if ( !cplx ) { // reorder
        if ( r2 < r1 ) std::swap( r1, r2 );
        if ( r1 < r0 ) std::swap( r0, r1 );
        if ( r2 < r1 ) std::swap( r1, r2 );
      }
      return;
    }
    /*              _
    ||  ___ __ __ _| |___
    || (_-</ _/ _` | / -_)
    || /__/\__\__,_|_\___|
    */
    // x^3 + aa * x^2 + bb * x + cc
    valueType aa = B/A;
    valueType bb = C/A;
    valueType cc = D/A;
    // scale Cubic Monic Polynomial
    valueType absa = std::abs(aa);
    valueType absb = std::sqrt(std::abs(bb));
    valueType absc = std::cbrt(std::abs(cc));

    indexType i_case = 0; // c MAX
    if ( absa < absb ) {
      if ( absc < absb ) i_case = 1; // |a| < |b| and |b| < |c| --> b MAX
      // |a| < |b| <= |c| --> c MAX
    } else {
      if ( absc < absa ) i_case = 2; // |b| <= |a| and |c| < |a| --> a MAX
      // |b| <= |a| < |c| --> c MAX
    }

    valueType scale(0), a[3];
    switch ( i_case ) {
    case 0:
      scale = absc;
      a[2]  = aa/absc;
      a[1]  = (bb/absc)/absc;
      a[0]  = cc > 0 ? 1 : -1;
      break;
    case 1:
      scale = absb;
      a[2]  = aa/absb;
      a[1]  = bb > 0 ? 1 : -1;
      a[0]  = ((cc/absb)/absb)/absb;
      break;
    case 2:
      scale = absa;
      a[2]  = aa > 0 ? 1 : -1;
      a[1]  = (bb/absa)/absa;
      a[0]  = ((cc/absa)/absa)/absa;
      break;
    }

    /*
    ||   __ _ _  _ ___ ______
    ||  / _` | || / -_|_-<_-<
    ||  \__, |\_,_\___/__/__/
    ||  |___/
    */
    // Class1: a[0] = −1, −1 <= a[1],a[2] <= +1
    // Class2: a[0] = +1, −1 <= a[1],a[2] <= +1
    // Class3: a[1] = −1, −1 <= a[0],a[2] <= +1
    // Class4: a[1] = +1, −1 <= a[0],a[2] <= +1
    // Class5: a[2] = −1, −1 <= a[0],a[1] <= +1
    // Class6: a[2] = +1, −1 <= a[0],a[1] <= +1
    indexType iclass = -1;
    switch ( i_case ) {
      case 0: iclass = a[0] > 0 ? 2 : 1; break;
      case 1: iclass = a[1] > 0 ? 4 : 3; break;
      case 2: iclass = a[2] > 0 ? 6 : 5; break;
    }
    bool use_shifted = false;
    trpx = false;
    switch ( iclass ) {
    case 1: r2 = guess1(a); break;
    case 2: r2 = guess2(a); break;
    case 3: r2 = guess3(a); break;
    case 4: r2 = guess4(a); break;
    case 5:
      r0   = a[1]-third;
      r1   = a[0]+one27th;
      trpx = std::abs(r0) <= machepsi && std::abs(r1) <= machepsi; // check for triple root
      if ( trpx ) { r0 = r1 = r2 = third * scale; nrts = 3; return; }
      use_shifted = std::abs(r0) <= 0.01 && std::abs(r1) <= 0.01;
      r2 = guess5(a);
      break;
    case 6:
      r0   = a[1]-third;
      r1   = a[0]-one27th;
      trpx = std::abs(r0) <= machepsi && std::abs(r1) <= machepsi; // check for triple root
      if ( trpx ) { r0 = r1 = r2 = -third * scale; nrts = 3; return; }
      use_shifted = std::abs(r0) <= 0.01 && std::abs(r1) <= 0.01;
      r2 = guess6(a);
      break;
    }

    /*
    ||          _
    ||  ___ ___| |_ _____
    || (_-</ _ \ \ V / -_)
    || /__/\___/_|\_/\___|
    */
    iter = 0;
    if ( use_shifted ) {
      if ( iclass == 5 ) {
        // a[2] == -1
        // y^3 + (a[1]-1/3)* y + (a[0]+a[1]/3-2/27), x = y+1/3
        r2 -= third; // shift guess
        iter = NewtonBisection( 0, r0, a[0]+a[1]/3-two27th, r2 );
        r2 += third; // unshift solution
      } else {
        // a[2] == 1
        // y^3 + (a[1]-1/3)* y + (a[0]-a[1]/3+2/27), x = y+1/3
        r2 += third; // shift guess
        r1 -= a[1]/3-one27th;
        //if ( std::abs(r3) < machepsi ) r3 = 0;
        iter = NewtonBisection( 0, r0, a[0]-a[1]/3+two27th, r2 );
        r2 -= third; // unshift solution
      }
    } else {
      iter = NewtonBisection( a[2], a[1], a[0], r2 );
    }

    // scale root
    r2 *= scale;

    /*
    // deflate
    // x^3 + aa*x^2 + bb*x + cc
    //    = (x-r2)*(x^2+b1*x+b0)
    //    = x^3 + x^2 * ( b1 - r2 ) + x * ( b0 - b1*r2 ) - r2 * b0
    //
    //    aa == b1 - r2
    //    bb == b0 - b1*r2
    //    cc == -r2 * b0
    //
    //  Solve the overdetermined linear system:
    //
    //  / 0    1  \            / aa + r2 \
    //  |         |  / b0 \    |         |
    //  | 1   -r2 |  |    |  = |   bb    |
    //  |         |  \ b1 /    |         |
    //  \ -r2  0  /            \   cc    /
    //
    //  if |r2| < 1 then solve
    //
    //  / 0    1  \  / b0 \    / aa + r2 \
    //  |         |  |    | =  |         |
    //  \ -r2  0  /  \ b1 /    \   cc    /
    //
    //  otherwise solve
    //
    //  / 1   -r2 \  / b0 \   / bb \
    //  |         |  |    | = |    |
    //  \ -r2  0  /  \ b1 /   \ cc /
    */
    valueType b0 = -cc/r2;
    valueType b1 = std::abs(r2) < 1 ? aa+r2 : -(cc/r2+bb)/r2;

    // solve quadratic polynomial
    Quadratic qsolve( 1.0, b1, b0 );
    nrts = qsolve.numRoots()+1;
    cplx = qsolve.complexRoots();
    dblx = qsolve.doubleRoot();
    r0   = qsolve.real_root0();
    r1   = qsolve.real_root1();

    if ( !cplx ) { // if real roots sort it!
      if ( r1 > r2 ) std::swap(r1,r2);
      if ( r0 > r1 ) std::swap(r0,r1);
      if ( r1 > r2 ) std::swap(r1,r2);
    }

  }

  void
  Cubic::info( std::ostream & s ) const {
    valueType const & A = ABCD[0];
    valueType const & B = ABCD[1];
    valueType const & C = ABCD[2];
    valueType const & D = ABCD[3];
    s << "\npoly a=" << A << " b=" << B << " c=" << C << " d=" << D
      << "\nn. roots = " << nrts
      << "\ncomplex  = " << (cplx?"YES":"NO")
      << "\ntriple   = " << (trpx?"YES":"NO")
      << "\ndouble   = " << (dblx?"YES":"NO");
    if ( cplx ) {
      s << "\nx0 = (" << r0 << "," <<  r1 << ')'
        << "\nx1 = (" << r0 << "," << -r1 << ')';
      if ( nrts > 2 ) s << "\nx3 = " << r2;
    } else {
      if ( nrts > 0 ) s << "\nx0 = " << r0;
      if ( nrts > 1 ) s << "\nx1 = " << r1;
      if ( nrts > 2 ) s << "\nx2 = " << r2;
    }
    s << '\n';
  }

  bool
  Cubic::check( std::ostream & s ) const {
    valueType const & A = ABCD[0];
    valueType const & B = ABCD[1];
    valueType const & C = ABCD[2];
    valueType const & D = ABCD[3];
    bool ok = true;
    valueType epsi = 10 * ( std::abs(A) +
                            std::abs(B) +
                            std::abs(C) +
                            std::abs(D) ) * machepsi;
    if ( cplx ) {
      valueType z0 = std::abs(eval( root0() ));
      valueType z1 = std::abs(eval( root1() ));
      valueType z2 = std::abs(eval( root2() ));
      valueType zr = eval( real_root0() );
      s << "|p(r0)| = " << z0
        << "\n|p(r1)| = " << z1
        << "\n|p(r2)| = " << z2
        << "\np(real_part(r0)) = " << zr
        << '\n';
      ok = z0 < epsi && z1 < epsi && z2 < epsi;
    } else if ( nrts == 1 ) {
      valueType z0 = eval( real_root0() );
      s << "p(r0) = " << z0  << '\n';
      ok = std::abs(z0) < epsi;
    } else if ( nrts == 2 ) {
      valueType z0 = std::abs(eval( root0() ));
      valueType z1 = std::abs(eval( root1() ));
      s << "p(r0) = " << z0
        << "\np(r1) = " << z1
        << '\n';
      ok = std::abs(z0) < epsi && std::abs(z1) < epsi;
    } else if ( nrts == 3 ) {
      if ( cplx ) {
        complexType z0 = eval( root0() );
        complexType z1 = eval( root1() );
        complexType z2 = eval( root2() );
        s << "p(r0) = " << z0
          << "\np(r1) = " << z1
          << "\np(r2) = " << z2
          << '\n';
        ok = std::abs(z0) < epsi && std::abs(z1) < epsi && std::abs(z2) < epsi;
      } else {
        valueType z0 = eval( real_root0() );
        valueType z1 = eval( real_root1() );
        valueType z2 = eval( real_root2() );
        s << "p(r0) = " << z0
          << "\np(r1) = " << z1
          << "\np(r2) = " << z2
          << '\n';
        ok = std::abs(z0) < epsi && std::abs(z1) < epsi && std::abs(z2) < epsi;
      }
    }
    return ok;
  }

}

#endif

// EOF: PolynomialRoots-2-Cubic.cc
```

### Quick search

### Table of Contents

- Quartic Roots Solver
- C++ API

«
hide menu

menu
sidebar
»

### Navigation

- index
- toc
- Quartic Roots »
- Program Listing for File PolynomialRoots-2-Cubic.cc

© Copyright 2021, Enrico Bertolazzi.
Created using Sphinx 4.2.0.
